# Infection by Tickborne Bacterium *Candidatus* Midichloria Associated with First Trimester Pregnancy Loss, Tennessee, USA

**DOI:** 10.3201/eid3102.240870

**Published:** 2025-02

**Authors:** John Newman, Caitlin Hughes, Karen C. Bloch, Khalil J. Deveaux, Scott Allen, Thao T. Truong, Behzad Najafian, Abelardo C. Moncayo, Lili Tao, Joshua Lieberman, Hernán Correa

**Affiliations:** Vanderbilt University Medical Center, Nashville, Tennessee, USA (J. Newman, C. Hughes, K.C. Bloch, L. Tao, H. Correa); University of Washington School of Medicine, Seattle, Washington, USA (K.J. Deveaux, S. Allen, T.T. Truong, B. Najafian, J. Lieberman); Tennessee Department of Health, Nashville (A.C. Moncayo)

**Keywords:** vector-borne infections, tickborne, infection, miscarriage, first trimester pregnancy loss, Candidatus Midichloria, Midichloriaceae, Tennessee, USA, ticks

## Abstract

A previously healthy 26-year-old woman in middle Tennessee, USA, experienced a first trimester pregnancy loss after multiple tick bites. Histopathology, 16S rRNA sequencing, and electron microscopy examination of the products of conception revealed an infection by a bacterium within the *Candidatus* Midichloria genus.

Chromosomal aneuploidy accounts for most first trimester pregnancy losses, but infections are estimated to cause ≈15% of early miscarriages (*1*). Evidence supporting an association of tickborne bacterial infections with early pregnancy loss is scarce and largely limited to case reports and small case series. Tickborne diseases with anecdotal associations to early pregnancy loss include Lyme disease, babesiosis, rickettsial diseases, and ehrlichiosis (*2*,*3*).

*Candidatus* Midichloriaceae represents a family of intracellular bacterial organisms first identified in the ovaries of the *Ixodes ricinus* tick, the primary vector of Lyme disease in Europe. Phylogenetic analysis led to the proposal for *Candidatus* Midichloriaceae to be separated into a third family under the order Rickettsiales, distinct from Rickettsiaceae and Anaplasmataceae, but likely more akin to Anaplasmataceae (*4*). Some members of the family boast a unique intramitochondrial life cycle, demonstrating tropism for a growing number of nonhuman hosts, such as aquatic invertebrates, protists, and various farm animals with unknown pathogenic potential (*5*). Further, the presence of *Candidatus* Midichloria genus mitochondrii DNA in human serum has rarely been documented without evidence of pathogenic effects (*6*). In this study, we describe a case of early pregnancy loss after tick exposure and the subsequent detection of *Candidatus* Midichloria sp. within the products of conception.

## The Study

**Figure 1:**
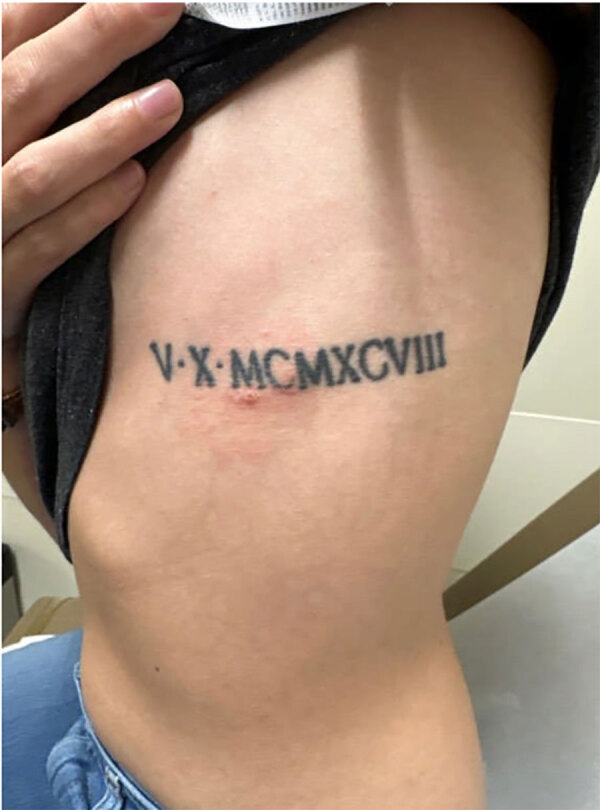
Left lateral torso rash with onset 2 weeks after tick removal in study of infection by tickborne bacterium *Candidatus* Midichloria associated with first trimester pregnancy loss, Tennessee, USA.

A 26-year-old woman in her first pregnancy sought care at 8 weeks’ gestation at an urgent care center in middle Tennessee, USA, with a 1-day history of a painful and pruritic rash on her torso at the site of a previous tick attachment (Figure 1). The rash consisted of a faint circular area of erythema surrounding a central shallow eschar. Two weeks before the rash developed, the patient removed 4 embedded ticks after walking her dogs in a field. The patient was unable to provide additional information regarding the physical characteristics of the ticks. The patient reported no systemic symptoms or recent domestic or international travel.

The patient was prescribed a 14-day course of cefuroxime axetil. Results of acute and convalescent serologic testing for *Ehrlichia chaffeensis*, *Borrelia burgdorferi*, and *Rickettsia rickettsii* infections were negative (Table). Results of complete blood count were unremarkable. After negative results of serologic testing were received, the patient was instructed to stop the prescribed antibiotic medications after ≈4 days of treatment.

**Table:** Laboratory testing at initial and follow-up evaluations in study of Infection by tickborne bacterium *Candidatus* Midichloria associated with first trimester pregnancy loss, Tennessee, USA*

Laboratory study	Acute testing, 2023 Apr 30, 2 weeks after tick bite	Convalescent testing, 2023 Jun 19, 11 weeks after tick bite
Complete blood count		
Leukocyte count, × 10^3^ cells/μL	7.4	7.8
Platelets, × 10^3^/μL	227	221
Liver function tests		
Alanine transaminase, U/L	Not evaluated	23
Aspartate transaminase, U/L	Not evaluated	24
Total bilirubin, mg/dL	Not evaluated	0.5
Alkaline phosphatase, U/L	Not evaluated	54
Serologic testing		
*Borrelia burgdorferi* Western blot		
IgM	0/3 bands	0/3 bands
IgG	2/10 bands	2/10 bands
*Ehrlichia chaffensis* EIA		
IgM	<1:16	<1:16
IgG	<1:64	<1:64
Spotted fever group *Rickettsia* EIA		
IgM	<1:16	<1:16
IgG	<1:64	<1:64
Routine prenatal infectious disease panel		
*Chlamydia trachomatis, Neisseria gonorrhoeae, Treponema pallidum*, HIV, hepatitis B virus, and hepatitis C virus	Negative	Not evaluated
Autoimmune panel		
Antinuclear antibody survey	Not evaluated	Positive 1:320, smooth pattern
SSA (Ro) IgG Qual	Not evaluated	Negative
SSA (La) IgG Qual	Not evaluated	Negative
Scl-70 IgG Qual	Not evaluated	Negative
Smith (Sm) IgG Qual	Not evaluated	Negative
RNP Ab Calc	Not evaluated	Negative
Anti-dsDNA	Not evaluated	Negative

*Anti-dsDNA, anti–double stranded DNA; EIA, enzyme immunoassay; RNP Ab Calc, anti–ribonucleoprotein antibody calculation; Scl-70 IgG Qual, scleroderma antibody qualitative.

The patient noted vaginal bleeding 2 weeks and 5 days after the urgent care visit; a subsequent prenatal ultrasound confirmed intrauterine fetal demise. The products of conception were routinely submitted for pathologic examination.

**Figure 2:**
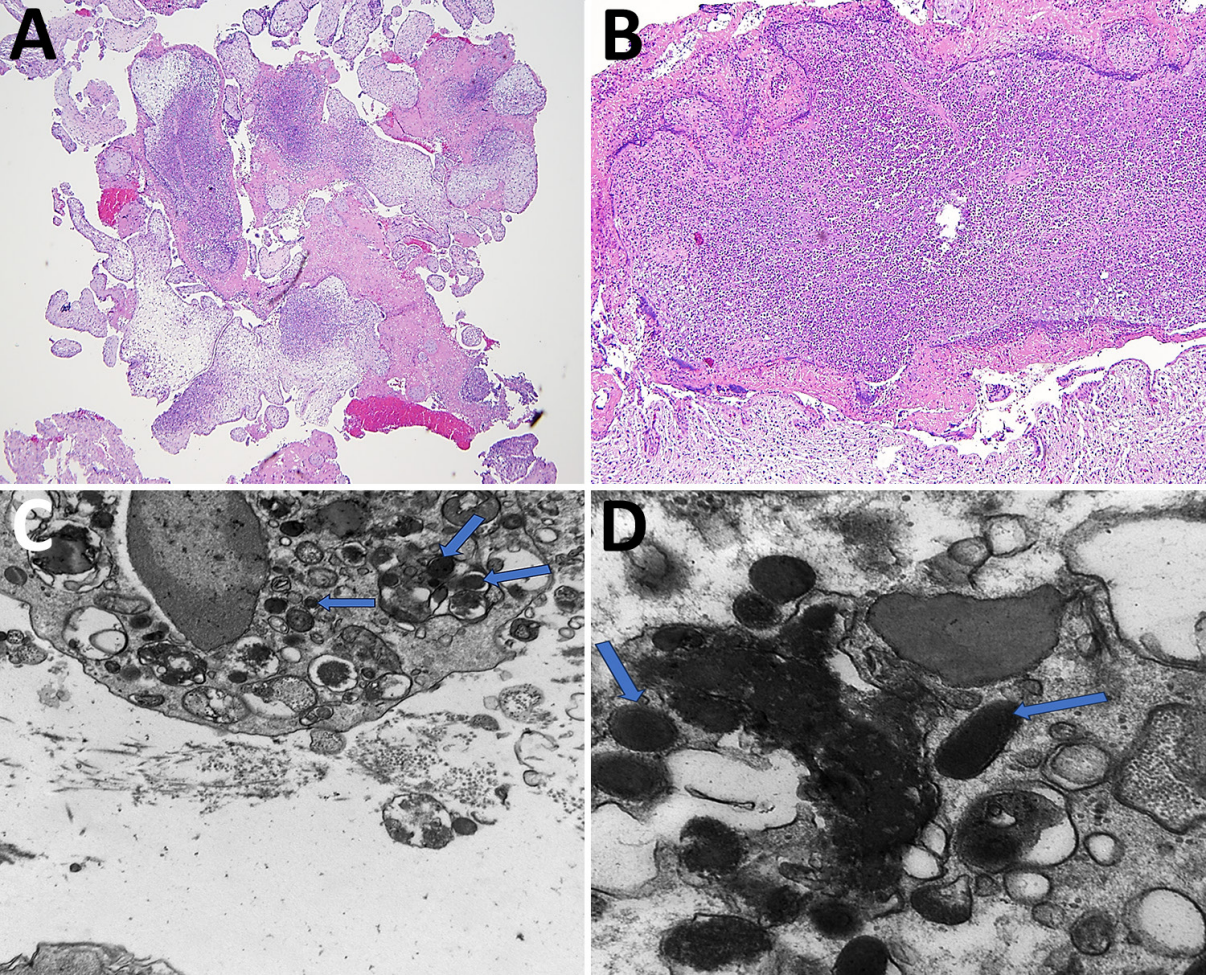
Imaging of samples from patient in study of infection by tickborne bacterium *Candidatus* Midichloria associated with first trimester pregnancy loss, Tennessee, USA. A, B) Formalin-fixed paraffin-embedded sections showing acute suppurative villitis and large intravillous abscesses. Original magnification ×40 for panel A and ×200 for panel B. C, D) Electron microscopy analysis was performed on tissue that was previously formalin-fixed but not paraffin-embedded. The formalin-fixed tissue was placed in a 2.5% glutaraldehyde solution before electron microscopy analysis. C) Intracellular bacterial forms in the cytosol (indicated by arrows) at ×20,000 magnification; D) cytoplasmic vacuoles (indicated by arrows) at ×60,000 magnification, measuring ≈0.25–0.34 μm × 0.40–0.53 μm.

Hematoxylin and eosin–stained sections showed marked acute villitis involving most of the trophoblastic villi. Large intravillous abscesses involved many villi. In addition, the acute inflammatory reaction appeared to originate at the villous tip and spread proximally to involve nearly the entire villous in a confluent fashion (Figure 2, panels A, B). Gram staining for bacteria and immunohistochemistry for cytomegalovirus were both negative. Giemsa special staining (Appendix Figure) showed darkly stained intracellular rods.

Electron microscopy examination of the trophoblastic villi demonstrated intracellular bacterial organisms within cytoplasmic vacuoles (Figure 2, panel C) and freely within the cytosol (Figure 2, panel D). The morphology of the organisms closely resembled that of the species *Candidatus* Midichloria mitochondrii, although definitive speciation cannot be rendered (*7*). Specifically, the organisms were round in cross-sectional profiles and appeared as short rods in longitudinal sections, measuring ≈0.25–0.34 µm in diameter and 0.40–0.53 µm in length. The bacteria exhibited an electron dense cytoplasm with vague granular content and an electron lucent outer membrane. Of note, the mitochondria were not involved in the sections examined.

After light microscopy examination, we submitted a formalin-fixed paraffin-embedded tissue block for a clinically available 16S rRNA broad range PCR, followed by Sanger sequencing (*8*). After primer and quality trimming, the resulting 297-bp product (Genbank accession no. PP102451) shared 95.9% nucleotide identity to *Candidatus* Midichloria mitochondrii strain IricVA (GenBank accession no. NC_015722) and 95.2% identity to another *I. ricinus*–associated 16S sequence (GenBank accession no. DQ788562). The identical sequence was subsequently detected in 2 replicate extractions from a second tissue block from the case. To assess for preanalytical contamination, we also tested 2 formalin-fixed paraffin-embedded blocks from different patients processed in the same batch as this case by broad-range bacterial PCR. Both blocks were negative for bacterial DNA, ruling out contamination during tissue processing.

**Figure 3:**
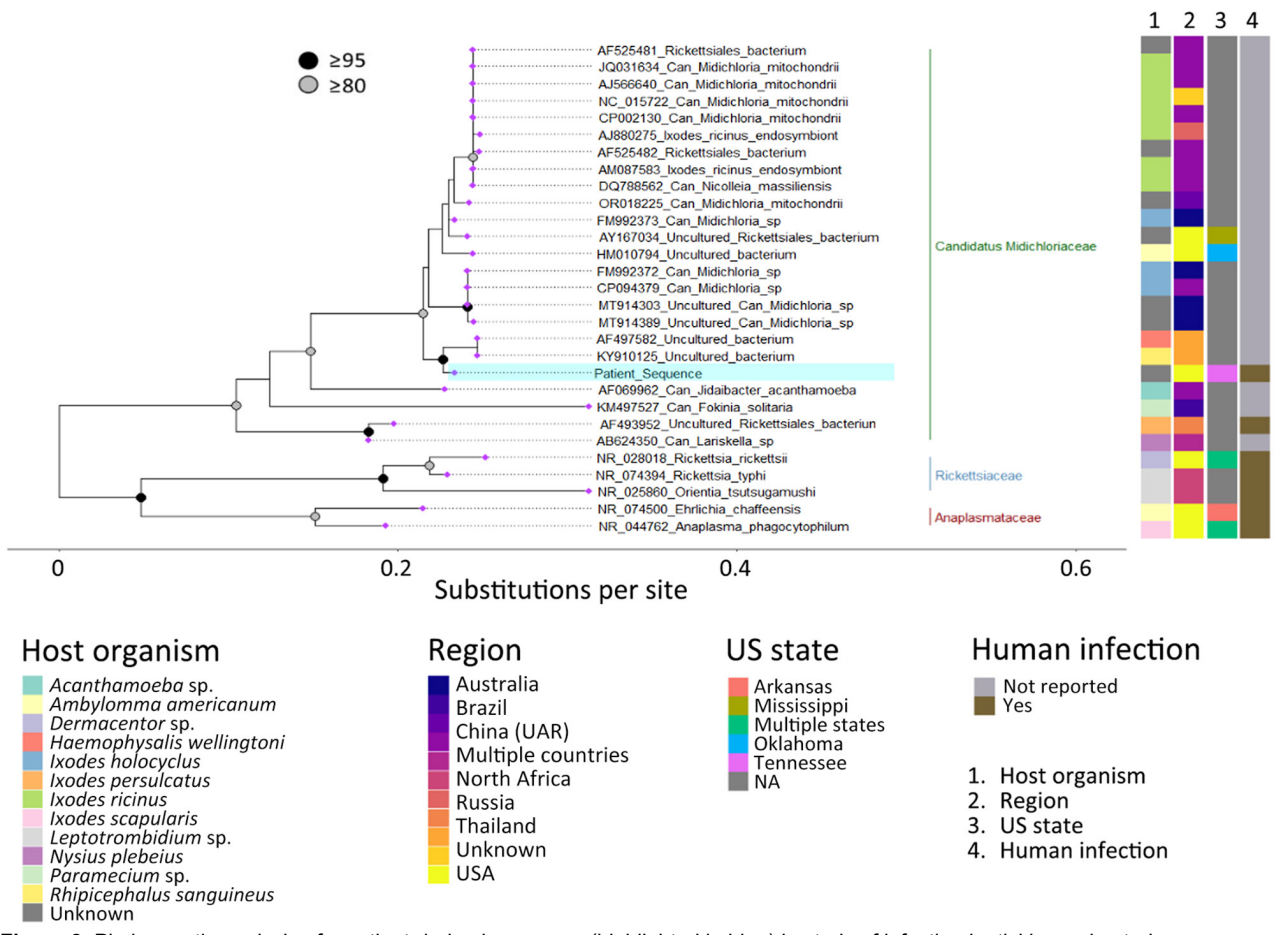
Phylogenetic analysis of a patient-derived sequence (highlighted in blue) in study of infection by tickborne bacterium *Candidatus* Midichloria associated with first trimester pregnancy loss, Tennessee, USA. Phylogenetic tree was generated using IQ-TREE (https://www.iqtree.org) with MAFFT-aligned (https://www.mafft.cbrc.jp/alignment/software) representative V1/V2 regions of 16S rRNA gene sequences from organisms within Rickettsiales and visualized with ggtree in R version 4.2.2 (The R Project for Statistical Computing, https://www.r-project.org). Sequences represented families Can. Midichloriaceae (green bar), Anaplasmataceae (red bar), and Rickettsiaceae (blue bar); most proximal sequences by BLAST analysis (https://blast.ncbi.nlm.nih.gov), including those used during clinical identification; and sequences previously associated with human specimens. GenBank accession numbers are indicated before the species name. Associated metadata indicate host tick, country reported, US state if applicable, and whether previously associated with a human infection. UAR, Uighur Autonomous Region. Nodes with >95% bootstrap support are in black, those with 80%–94.9% support are in gray. Tip labels are purple.

We analyzed the patient-derived 16S sequence in the context of representative Rickettsiales sequences (Figure 3). In the resulting tree, the detected bacterial sequence had the highest homology with a sequence from Thailand (accession no. KY910125) and was in a clade with *Candidatus* Midichloria spp. (80%–90% bootstrap support). The detected sequence was distinct from *Candidatus* M. mitochondrii, as well as other endosymbionts within the genus including *Candidatus* Lariskella sp. (AB624350).

## Conclusions

*Candidatus* Midichloriaceae are proposed as a separate family under the Rickettsiales order, but few studies have attempted to describe the biology of this relatively unexplored region of the bacterial taxonomy. Bacteria of this family have been isolated in numerous tick vectors known to transmit pathogenic bacteria, including members of the *Ixodes*, *Rhipicephalus*, *Amblyomma*, *Hyalomma*, and *Dermacentor* genera, and have the capacity for mammalian inoculation (*9*). *Candidatus* Midichloriaceae organisms have a predilection for female ticks and demonstrate tropism for the salivary glands and ovaries, enabling vertical transmission to tick progeny (*10*). Evidence suggests at least facultative mutualism between the tick host and some *Candidatus* Midichloriaceae organisms; *Candidatus* M. mitochondrii has been shown to increase the tick’s feeding ability during blood meals, as well as aid in the production of superior larvae (*11*,*12*).

The pathogenic potential of *Candidatus* Midichloriaceae in intermediate hosts is incompletely understood. Three studies have reported isolating antibodies to *Ca.* Midichloriaceae or microbial DNA from the blood of human hosts exposed to tick bites, with similar detection rates as a unique organism or in combination with another tickborne pathogen (*6*,*13*,*14*). Those results suggest that *Candidatus* Midichloriaceae antigens or nucleic acids can be transmitted to humans and used as a marker for tick bites but do not demonstrate independent pathogenicity or replicative ability. One of the 3 studies potentially identified a bacterium from the *Candidatus* Midichloriaceae family causing a mild febrile illness in east Russia in 2004 (*14*). Our results suggest a strong association between infection by *Candidatus* Midichloria sp. and the pregnancy loss in this patient.

Although electron microscopy images showed intracellular bacteria that could be compatible with *Candidatus* Midichloria sp., this study is limited by the lack of clear internal structures, although vague granular content suggestive of organelles was present. Therefore, definitive identification cannot be rendered. The only other known pathogen on the histologic differential diagnosis is *Listeria monocytogenes*, which classically displays villous microabscesses. However, the abscesses caused by *L. monocytogenes* are smaller, and the acute inflammation does not tend to be confluent or span the entire length of the villous (*15*). Further, 16S rRNA sequencing did not detect *L. monocytogenes* in 2 replicate extractions, and *L. monocytogenes* organisms are typically situated in phagolysosomes and measure 500–2,000 nm in length.

This case describes an early pregnancy loss associated with an organism within the *Candidatus* Midichloriaceae family. This finding suggests tropism for human trophoblastic tissue and potentially deleterious effects for the fetus. Although further studies are needed to elucidate the taxonomy, hosts, life cycle, and pathogenesis of *Candidatus* Midichloria sp. and related endosymbionts, clinicians and patients should be aware of this emerging pathogen. Further, for pregnant patients with tick bites, treatment with doxycycline should be considered because the benefits could outweigh the risks. Pregnant patients, especially those in the southeastern United States, should be counseled on the risk for tick exposure during early pregnancy, as well as on proper tick removal technique.

Appendix Additional information about infection by tickborne bacterium *Candidatus* Midichloria associated with first trimester pregnancy loss, Tennessee, USA.
