# Supplementary material for: Infection by Tickborne Bacterium Candidatus Midichloria Associated with First Trimester Pregnancy Loss, Tennessee, USA
Source: Emerg Infect Dis. 2025 Feb;31(2):350–4. doi: 10.3201/eid3102.240870 (PMC11845134; doi:10.3201/eid3102.240870)
Supplement: Appendix — Additional information about infection by tickborne bacterium Candidatus Midichloria associated with first trimester pregnancy loss, Tennessee, USA. [file 24-0870-Techapp-s1.pdf]

Article DOI: https://doi.org/10.3201/eid3102.240870

EID cannot ensure accessibility for supplementary materials supplied by authors. Readers who have difficulty accessing supplementary content should contact the authors for assistance.

Infection by Tickborne Bacterium *Candidatus* Midichloria Associated with First Trimester Pregnancy Loss, Tennessee, USA

Appendix

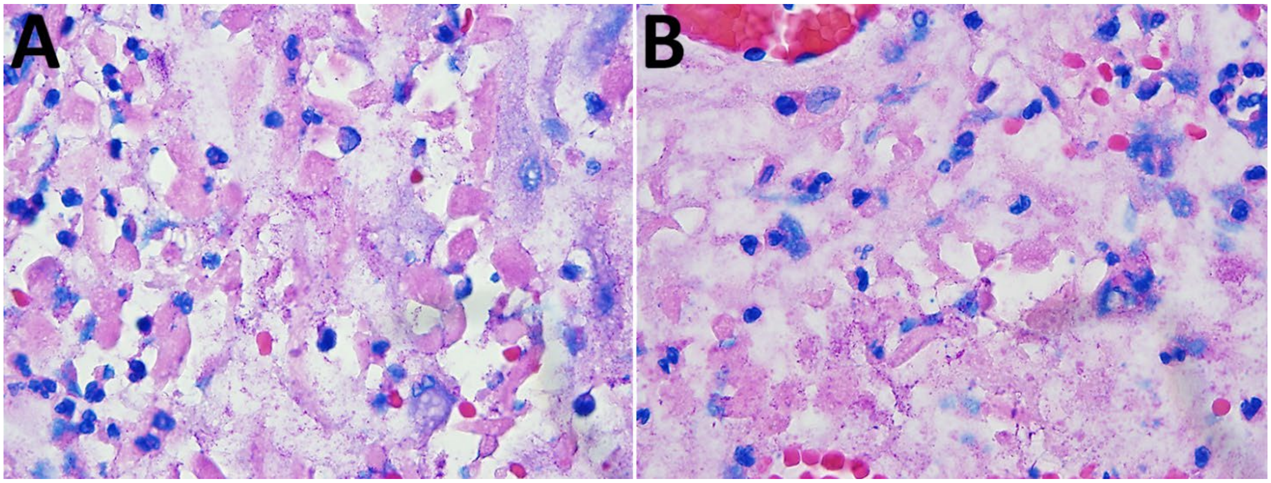

Appendix Figure. Giemsa special staining reveals darkly stained, small, intracellular rod-shaped structures within stromal fibroblasts and macrophages (A-B, 1000X under immersion oil).
